# Comparative Study of the Magnetic Behavior of FINEMET Thin Magnetic Wires: Glass-Coated, Glass-Removed, and Cold-Drawn

**DOI:** 10.3390/ma16041340

**Published:** 2023-02-04

**Authors:** Sorin Corodeanu, Costică Hlenschi, Horia Chiriac, Tibor-Adrian Óvári, Nicoleta Lupu

**Affiliations:** National Institute of Research and Development for Technical Physics, 700050 Iași, Romania

**Keywords:** thin magnetic wires, magnetic properties, magnetic permeability, magnetoimpedance, stresses, annealing

## Abstract

In this paper, a comparative investigation of the magnetic behavior and its stress dependence in the case of FINEMET glass-coated, glass-removed, and cold-drawn microwires at low and high frequencies, respectively, is presented. The experimental results show major differences between their magnetic properties depending on the preparation method and microwire diameter. The evolution of the magnetic permeability, coercivity, and magnetoimpedance responses with the applied tensile force was investigated and analyzed in correlation with the stresses induced during preparation, their relief following annealing, and the annealing-induced structural transformations. The coercivity dependence on applied force was found to show the highest sensitivity in the glass-removed microwires, while the magnetic permeability and magnetoimpedance sensitivity to force were found to be higher in the cold-drawn samples. The results of this comparative study will enable an enhanced material selection process for various applications in miniaturized magnetic and stress sensors with increased sensitivity.

## 1. Introduction

Currently, Fe-rich microwires are of great interest for both basic research and applications due to their excellent magnetic and magnetoelastic properties [1,2,3,4,5]. A comprehensive understanding of the dependence of the magnetoelastic properties of Fe-rich microwires on different external factors (applied magnetic field, force, and torque) would significantly facilitate the take-up of these materials as sensing elements in various types of sensors [6,7,8]. The magnetic properties of the thin amorphous microwires are affected by the magnetostriction coefficient due to their coupling with internal and external stresses [5,8,9,10]. Lower magnetostriction values and superior soft magnetic properties can be achieved by means of structural changes, such as the nanocrystalline phase formation in an Fe-rich amorphous precursor with small additions of Cu and Nb after annealing at temperatures between 500 °C and 600 °C [3,4,5,6,7]. The nanocrystalline alloy patented under the trade name FINEMET [11] is likely the most well-known and studied in this class. This nanocrystalline alloy was found to show lower magnetostriction values (~10^−6^), a very high value of the relative permeability (higher than 10^5^), very low coercivity (lower than 1 A/m), and high saturation polarization (~1.2 T) [11,12,13,14,15,16]. Compared with other soft magnetic materials, such as those from the Co-Fe-Si-B system, with very small negative magnetostriction, the FINEMET alloy presents the advantage of even higher magnetization and permeability, lower coercivity [5,6,7], and, as we will show in this study, a highly sensitive stress dependence of the magnetic properties, which is very useful for developing stress-sensing related applications.

A unique process that consists in the formation of nanosized crystalline grains and their controlled subsequent growth is at the origin of such outstanding magnetic and magnetoelastic properties. This process is driven by annealing at suitable temperatures for well-determined time intervals and is made possible by the well-defined composition, more specifically by the Cu and Nb additions to the base Fe-Si-B alloy. Thus, optimum annealing will result in the formation and growth of α-Fe(Si) crystalline grains with sizes from several nm up to about 20 nm. These nanograins are randomly spread throughout the residual amorphous matrix. When the size of the grains and their density reach certain threshold values, they will interact through exchange coupling and then behave as a single phase. Since the magnetostriction of the residual amorphous matrix and that of the crystalline grains phase have opposite signs and different values, the overall magnetostriction of the alloy wires will be averaged out to very small values [7]. The actual value of the global magnetostriction is influenced by the volume fractions of the two phases. The various magnetic anisotropies in different types of wires also play an important role in defining the value of the exchange length in each case, i.e., the distance below which the nanosized grains will be exchange coupled [3].

The aim of this paper is to present a comparative study of the magnetic properties of three types of thin FINEMET magnetic microwires: glass-coated, glass-removed, and cold drawn, as well as of their applied stress dependence, regarding their potential use in low-field and stress sensing applications, such as low magnetic field detectors, acoustic sensors, pressure gauges, force sensors, etc. Although their excellent soft magnetic properties and unique magnetic behavior are suitable for such applications, one expects a few significant differences among the three wire types, arising from their different preparation methods and diameters.

## 2. Materials and Methods

**Figure 1:**
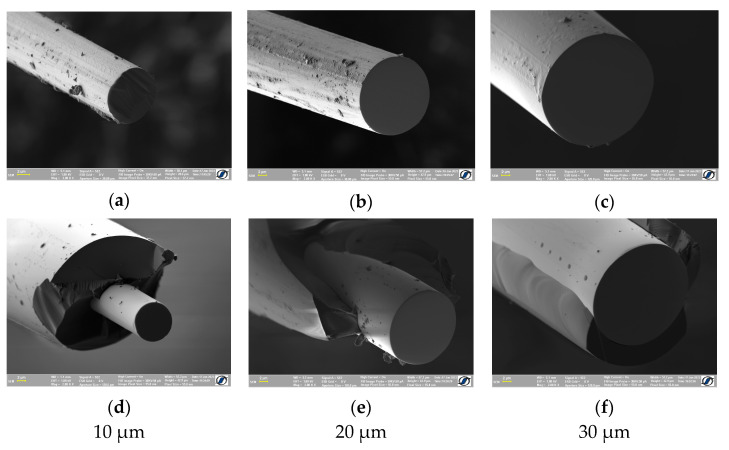
Scanning Electron Microscopy (SEM) images of cold-drawn (**a**–**c**) and glass-coated (**d**–**f**) Fe_73.5_Si_13.5_B_9_Cu_1_Nb_3_ microwires having 10, 20, and 30 µm metallic core diameters.

Three types of FINEMET (Fe_73.5_Si_13.5_B_9_Cu_1_Nb_3_) samples were analyzed in this study: as-cast glass-coated, glass-removed, and cold-drawn microwires (Figure 1). The cold-drawn microwires with diameters of 30 µm, 20 µm, and 10 µm, respectively, were prepared by successively cold-drawing an amorphous wire with a starting diameter of 105 µm, which had previously been obtained by in-rotating-water melt spinning [17]. The glass-coated thin samples with metallic wire diameters of 30 µm, 20 µm, and 10 µm, respectively, and a total diameter ranging between 34 and 40 µm, were prepared using the glass-coated melt spinning technique [18]. The so-called “glass-removed” microwires were prepared by mechanically removing the glass coating from the as-cast glass-coated microwires by pressing the wire between a flat surface and a rotating cylinder. All the thin wire samples employed in this investigation were prepared using the facilities at the National Institute of R&D for Technical Physics in Iași, Romania.

The initial, precursor wires, were all in amorphous state and were prepared by means of the two above-mentioned rapid quenching processes: in-rotating-water melt spinning and glass-coated melt spinning. Further post-production processing, such as cold drawing and glass removal, was applied to the as-quenched wires. The two rapid solidification techniques resulted in cylindrical wires with different dimensions and overall characteristics. For instance, in-rotating-water melt spinning allows the preparation of amorphous wires with diameters around 100 μm, whereas glass-coated melt spinning results in composite samples—a metallic nucleus within a Pyrex glass coating, with significantly smaller dimensions: metallic nucleus diameters below 50 μm and glass coating thickness below 30 μm. This leads to different distributions and levels of the mechanical stresses induced by the rapid quenching of the melt. Moreover, in the case of glass-coated samples, there are also stresses induced by the glass coating. Cold drawing induces extra stresses in the wires, while glass removal will relieve some of the initial stresses. This information is very important, since the initial state of the wire samples that were subsequently annealed in order to induce the nanocrystalline phase formation depends on all these steps, starting with the specific preparation method and additional processing, e.g., cold drawing and glass removal. Hence, annealing is expected to produce different results.

All the samples were annealed in vacuum for 1 h at temperatures between 300 °C and 600 °C for two reasons: (1) to reduce the intrinsic mechanical stresses induced during their preparation and (2) to initiate the formation of the optimum nanocrystalline structure, specific to FINEMET alloys [15,19].

The coercivity and relative magnetic permeability were measured in the longitudinal (axial) direction using a modified a.c. fluxmetric method [20], under applied magnetic fields up to 30 kA/m at 50 Hz. For the magnetoimpedance (MI) characterization, the samples’ impedance was measured using an Agilent E4991A impedance analyzer at frequencies of the a.c. driving current between 10 and 250 MHz. Both characterization techniques were adapted to allow the study of magnetic behavior under applied mechanical stresses. The experiments on samples subjected to tensile forces were performed by attaching specific weights to one end of the sample while the other end was fixed. The applied force was calculated as *F* = *m*·*g*, where *m* is the actual mass attached to the sample end and *g* ≅ 9.8 m/s^2^ is the gravitational acceleration.

## 3. Results and Discussion

**Figure 2:**
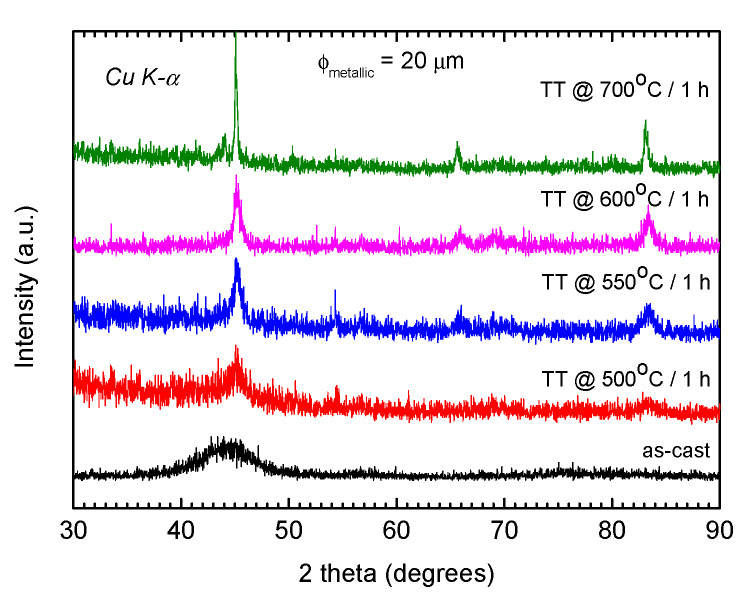
The evolution of the microstructure with the annealing temperature for glass-removed Fe_73.5_Si_13.5_B_9_Cu_1_Nb_3_ microwires having 20 µm metallic core diameter. The optimum annealing temperature range is between 500 and 600 °C, where the nanocrystal size varies between 10 and 20 nm.

The evolution of the samples’ microstructure was investigated by means of X-ray diffraction (XRD). All the samples in the as-cast state are fully amorphous, while the annealing between 500 and 600 °C for 1 h is responsible for the appearance of the α-Fe(Si) nanosized crystalline grains of various sizes and concentrations, in agreement with previous results [3,15]. For instance, Figure 2 presents the X-ray diffraction patterns for glass-removed FINEMET microwires with 20 µm in diameter after different stages of annealing. Similar results were observed for cold-drawn and glass-coated microwires, irrespective of their diameter.

**Figure 3:**
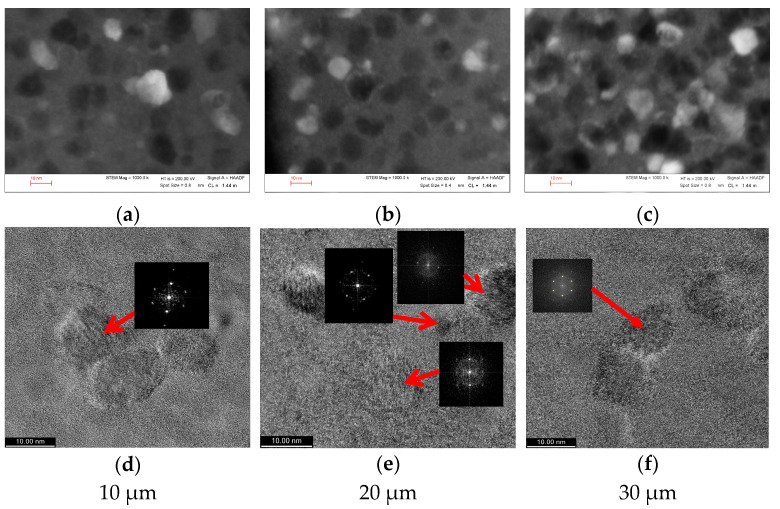
(**a**–**c**) The evolution of the grain sizes and density for cold-drawn Fe_73.5_Si_13.5_B_9_Cu_1_Nb_3_ microwires with various diameters, annealed at 500 °C for 1 h. (**d**–**f**) The UHR-TEM and SAED images of the same samples indicate similar bcc α-Fe(Si) grain sizes (between 10 and 15 nm), randomly oriented.

XRD results and Transmission Electron Microscopy (TEM) images both show that all the samples exhibit similar evolutions of their microstructures with annealing, irrespective of their preparation methods or diameters (Figure 3). Any differences that appear are ascribed to the different levels and distribution of residual internal stresses that affect the magnetic anisotropy and, consequently, the value of the exchange length below which the nanosized grains interact. This interaction is influenced by the number and density of the bcc α-Fe(Si) nanograins, which vary with the sample diameter, as shown by the STEM images in Figure 3a–c. However, it is important to note that the size and structure of the nanocrystals are similar, irrespective of the microwire diameter, as indicated by the ultra-high resolution TEM images and selected area electron diffraction (SAED) patterns (Figure 3d–f).

**Figure 4:**
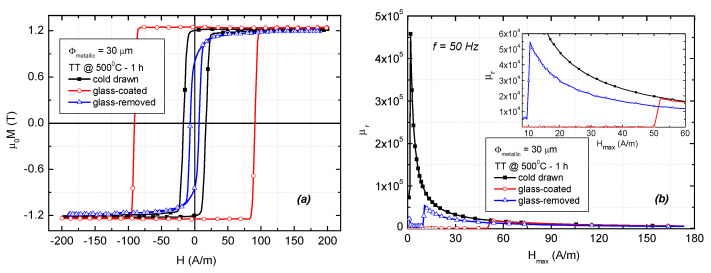
(**a**) Axial hysteresis loops and (**b**) variation of relative magnetic permeability with applied magnetic field for annealed (500 °C—1 h) Fe_73.5_Si_13.5_B_9_Cu_1_Nb_3_ microwires having 30 µm metallic core diameter. (Sample length: l_s_ = 10 cm).

The axial magnetization curves and the variation of the relative magnetic permeability versus applied magnetic field in the case of the glass-coated, glass-removed, and cold-drawn nanocrystalline microwire samples having an actual metallic wire diameter of 30 µm, are shown in Figure 4. All measurements were performed on 10 cm long samples using a driving field frequency of 50 Hz. The measurements were performed on samples annealed for 1 h at 500 °C, which exhibit the highest magnetic permeability and significant stress dependence, in agreement with previous reports [15,19].

The cold-drawn and the glass-coated microwires present a magnetically bistable behavior, characterized by a rectangular shape of the hysteresis loops, also called the large Barkhausen effect, which shows that magnetization reversal takes place through the depinning and propagation of a single domain wall. After removing the glass from the glass-coated microwires, the hysteresis loop is no longer rectangular, with the magnetization increasing gradually with the applied magnetic field, which indicates the presence of magnetization rotation processes.

The bistable magnetic behavior vanishes due to the relaxation of the internal stresses induced previously by the glass coating. The value of the coercivity is smaller (less than 20 A/m) for the samples without glass coating (cold-drawn and glass-removed) and much higher (~90 A/m) for the glass-coated microwire, due to the high stress induced by the coating [21].

The maximum relative magnetic permeability shows considerably higher values for the cold-drawn microwires in comparison with the glass-coated and glass-removed ones and has larger values for thicker samples, the largest being in the case of the cold-drawn microwire with 30 µm in diameter (4.6 × 10^5^ at a maximum applied field of 1.7 A/m).

**Figure 5:**
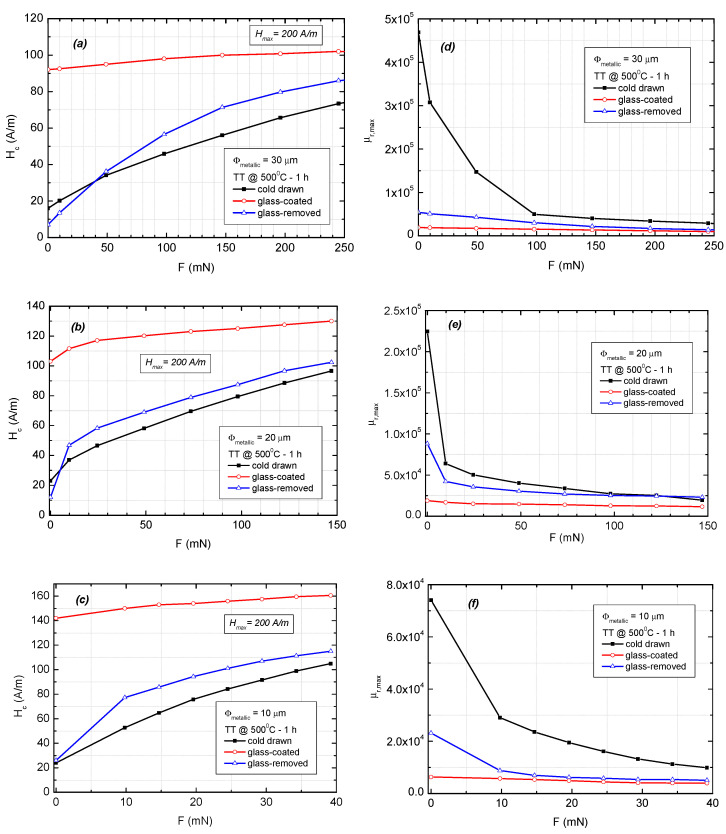
Variation of coercivity (**a**–**c**) and maximum value of the magnetic permeability (**d**–**f**) versus applied tensile force, measured on the annealed (500 °C—1 h) Fe_73.5_Si_13.5_B_9_Cu_1_Nb_3_ cold-drawn, glass-coated, and glass-removed microwires, with 30 µm, 20 µm, and 10 µm metallic core diameters, respectively. Sample length: l_s_ = 10 cm.

In order to find the most suitable material for stress-sensing-related applications, we compared the results of the experiments performed under an applied tensile force on the different types of magnetic wires. The evolution of the coercivity and maximum permeability values versus longitudinally applied force is illustrated in Figure 5. The coercivity values were extracted from the hysteresis loops measured using a longitudinal excitation magnetic field with an amplitude of 200 A/m. The maximum permeability values were extracted from the relative permeability dependence on the amplitude of the excitation field.

In the case of glass-coated microwires, the coercivity and permeability values exhibit a rather weak dependence on the applied tensile force, mainly since the magnetic wires are already subjected to the high intrinsic tensile stresses induced by the glass coating [22,23]. For microwire samples without glass (cold-drawn and glass-removed), the value of the coercivity increases monotonically with the applied tensile force. The microwires with the removed glass coating having a diameter of 10 μm display the highest coercivity variation as a function of the applied tensile force, i.e., 5.2 (A/m)/mN for low applied force values (see Figure 5c).

When no stress is applied, the maximum values of the relative magnetic permeability are considerably higher for the cold-drawn samples as compared with those for the glass-coated and glass-removed ones. When axial tension is applied, the corresponding axial magnetic permeability exhibits a sharp drop for very low force values (see Figure 5d–f). The permeability decreases more rapidly for low applied forces in the case of the cold-drawn wires as compared with the glass-coated and glass-removed ones. The measured sensitivity to applied force of the relative magnetic permeability was around 16,400 mN^−1^ for the cold-drawn microwires with 20 μm and 30 μm in diameter, respectively, and 4600 mN^−1^ for the same type of microwires having a diameter of just 10 μm, for applied forces below 10 mN.

The stress-dependent properties at high frequencies were investigated by means of magnetoimpedance (MI) effect measurements at a constant current of 1 mA and a working frequency of 100 MHz on nanocrystalline samples having a length of 15 mm. For these measurements, we employed samples annealed for 1 h at 550 °C, in which case we obtained the largest relative variation of the impedance, as it was previously reported [15,19]. This is mainly caused by the distribution of the stresses, namely the larger stresses in the near surface region [15], which require slightly higher annealing temperatures (550 °C) to achieve the maximum magnetoimpedance response, compared with the 500 °C necessary to reach the lowest coercivity and maximum permeability.

**Figure 6:**
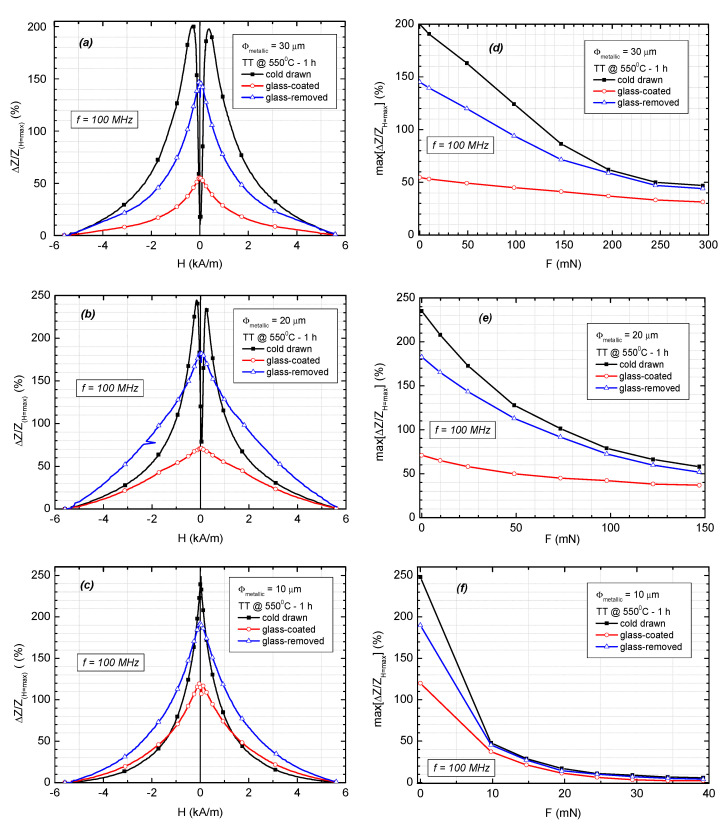
Relative variation of the impedance versus axially applied magnetic field (**a**–**c**) and its maximum value versus applied tensile force (**d**–**f**), measured on annealed (550 °C—1 h) Fe_73.5_Si_13.5_B_9_Cu_1_Nb_3_ cold-drawn, glass-coated, and glass-removed microwires, with 30 µm, 20 µm, and 10 µm metallic core diameters, respectively. Sample length, l_s_ = 15 mm.

The dependence of the relative variation of the impedance and its maximum on the applied magnetic field and on the applied tensile force, respectively, are illustrated in Figure 6. The cold-drawn microwires present the highest MI ratio, with double peak behavior for the wires with 20 µm and 30 µm in diameter and single peak behavior for the one with 10 µm in diameter. For the glass-coated microwires with low negative or positive magnetostriction, such as the CoFeSiB amorphous ones, the high impedance variation versus applied field originates in the existence of a core-shell magnetic structure within the magnetic wire, with the inner core being axially magnetized and the outer shell having a circumferential magnetization [22]. The outer shell with circumferential anisotropy appears as a result of the coupling between their low negative magnetostriction and the large internal stresses induced by the glass coating and by externally applied tensile forces [22,23,24]. The double peak behavior appears when the internal tensile stress is large and, consequently, the volume of the region with circumferential anisotropy is large, while the single peak behavior is caused by the axial anisotropy of the central part of the magnetic wire [22,23,24].

For the materials showing small positive magnetostriction, such as those prepared from the FINEMET alloy, the double peak behavior appears in the microwires that were annealed under tension due to so-called “back stress,” which acts as a compressive longitudinal stress [25,26]. The compressive stress coupled with low positive magnetostriction is expected to lead to a similar core-shell magnetic structure with circular anisotropy in the outer shell. The compressive stresses generated within the magnetic wire during the cold-drawing process are very strong, therefore generating significant residual stresses even after annealing in the microwires with larger diameters, which present a double peak MI behavior, and less so in the thinner ones, e.g., the 10 µm sample.

For the glass-coated wires, the MI response is low due to the high axial tensile stresses induced by the glass coating, in agreement with previous results [21].

By applying axial tensile stresses to the magnetic wires, the amplitudes of the MI curves decrease with the increase in the tensile forces for all the analyzed samples (see Figure 3d–f). The MI amplitude decreases faster for low applied forces, also in the case of the cold-drawn wires. The measured sensitivity to force of the MI amplitude is 20%·mN^−1^ for the cold-drawn wires with 10 μm in diameter, 3%·mN^−1^ for the cold-drawn wires with 20 μm in diameter, and just 1%·mN^−1^ for the same type of wires having a diameter of 30 μm, for applied forces below 10 mN. The high sensitivity of the 10 μm cold-drawn wires to applied force makes them suitable for applications such as sensing elements in magneto-mechanical sensors for the detection of very small mechanical stresses.

## 4. Conclusions

A comparative study of the magnetic behavior and properties at low and high frequencies of three types of FINEMET thin microwires—glass-coated, glass-removed, and cold-drawn—alongside their dependence on axial tensile forces was performed.

The results show significant differences among their magnetic properties and behavior, which originate both in their different preparation techniques as well as in the different diameters of the various samples. The highest relative magnetic permeability, 4.6 × 10^5^, and the lowest coercivity, 12 A/m, were measured in the case of a cold-drawn microwire having 30 µm in diameter, in the absence of any applied stress. The magnetoimpedance response also presents higher values in the case of cold-drawn microwires, with a maximum of 248% variation for thinner samples, i.e., for wires with 10 µm in diameter.

When a tensile force is applied to such wires, their magnetic properties suffer deterioration: coercivity increases, while the relative magnetic permeability and the magnetoimpedance response are both decreasing. The sensitivity to force of the glass-removed wires exhibits the highest coercivity dependence on the applied force, with a maximum of 5.2 (A/m)/mN for a sample having 10 µm in diameter. The largest permeability variation to applied forces was observed in the case of cold-drawn wires, with a maximum of 16,400 mN^−1^ for those with 20 µm and 30 µm in diameter. The sensitivity to force of the magnetoimpedance response was also higher for cold-drawn samples, with a maximum of 20%·mN^−1^ for cold-drawn wires having 10 µm in diameter.

The superior soft magnetic properties of the investigated materials recommend them for applications as sensitive elements in different types of magnetic sensors, while the important dependence of their magnetic properties on low applied tensile forces could be used to develop novel stress/force-related sensors.

## Data Availability

The data that support the findings of this study are available from the corresponding authors, upon reasonable request.

## References

[1] Morón C., Cabrera C., Morón A., García A., González M. (2015). Magnetic sensors based on amorphous ferromagnetic materials: A review. Sensors.

[2] Zhukov A., Ipatov M., Churyukanova M., Kaloshkin S., Zhukova V. (2014). Giant magnetoimpedance in thin amorphous wires: From manipulation of magnetic field dependence to industrial applications. J. Alloys Compd..

[3] Chiriac H., Lupu N., Stoian G., Ababei G., Corodeanu S., Óvári T.-A. (2017). Ultrathin nanocrystalline magnetic wires. Crystals.

[4] Churyukanova M., Zhukova V., Kaloshkin S., Zhukov A. (2012). Effect of magnetoelastic anisotropy on properties of Finemet-type microwires. J. Alloys Compd..

[5] Phan M.H., Peng H.X. (2008). Giant magnetoimpedance materials: Fundamentals and applications. Prog. Mater. Sci..

[6] Mohri K., Humphrey F.B., Panina L.V., Honkura Y., Yamasaki J., Uchiyama T., Hirami M. (2009). Advances of amorphous wire magnetics over 27 years. Phys. Status Solidi A.

[7] Herzer G., Vazquez M., Knobel M., Zhukov A., Reininger T., Davies H.A., Grossinger R., Sanchez J.L. (2005). Round table discussion: Present and future applications of nanocrystalline magnetic materials. J. Magn. Magn. Mater..

[8] Garcia C., Chizhik A., Zhukov A., Zhukova V., Gonzalez J., Blanco J.M., Panina L.V. (2007). Influence of torsion and tensile stress on magnetoimpedance effect in Fe-rich amorphous microwires at high frequencies. J. Magn. Magn. Mater..

[9] Chiriac H., Óvári T.-A., Zhukov A. (2003). Magnetoelastic anisotropy of amorphous microwires. J. Magn. Magn. Mater..

[10] Santos J.D., Olivera J., Álvarez P., Sánchez T., Pérez M.J., Sánchez M.L., Gorría P., Hernando B. (2007). Torsion-induced magnetoimpedance in nanocrystalline Fe-based wires. J. Magn. Magn. Mater..

[11] Yoshizawa Y., Oguma S., Yamauchi K. (1988). New Fe-based soft magnetic alloys composed of ultrafine grain structure. J. Appl. Phys..

[12] Herzer G., Buschow K.H.J. (1997). Nanocrystalline Soft Magnetic Alloys. Handbook of Magnetic Materials.

[13] Herzer G. (2002). Grain size dependence of coercivity and permeability in nanocrystalline ferromagnets. IEEE Trans. Magn..

[14] Allia P., Baricco M., Tiberto P., Vinai F. (1993). Kinetics of the amorphous to nanocrystalline transformation in Fe73.5Cu1Nb3Si13.5B9. J. Appl. Phys..

[15] Chiriac H., Corodeanu S., Donac A., Dobrea V., Ababei G., Stoian G., Lostun M., Óvári T.-A., Lupu N. (2015). Influence of cold drawing on the magnetic properties and giant magneto-impedance response of FINEMET nanocrystalline wires. J. Appl. Phys..

[16] Hernando B., Olivera J., Sanchez M.L., Prida V.M., Varga R. (2008). Temperature dependence of magnetoimpedance and anisotropy in nanocrystalline Finemet wire. IEEE Trans. Magn..

[17] Ogasawava I., Ueno S. (1995). Preparation and properties of amorphous wires. IEEE Trans. Magn..

[18] Chiriac H., Óvári T.-A. (1996). Amorphous glass-covered magnetic wires: Preparation, properties, applications. Prog. Mater. Sci..

[19] Donac A., Corodeanu S., Lupu N., Óvári T.-A., Chiriac H. (2016). Magnetic properties and giant magnetoimpedance in FINEMET cold drawn microwires. J. Optoelectron. Adv. Mater.—Rapid Commun..

[20] Corodeanu S., Chiriac H., Lupu N., Óvári T.-A. (2011). Magnetic characterization of submicron wires and nanowires using digital integration techniques. IEEE Trans. Magn..

[21] Chiriac H., Óvári T.-A., Pop G., Barariu F. (1997). Magnetic behavior of nanostructured glass covered metallic wires. J. Appl. Phys..

[22] Vazquez M., Garcia-Beneytez J.M., Garcia J.M., Sinnecker J.P., Zhukov A.P. (2000). Giant magneto-impedance in heterogeneous microwire. J. Appl. Phys..

[23] Chiriac H., Óvári T.A., Pop G. (1995). Internal stress distribution in glass-covered amorphous magnetic wires. Phys. Rev. B.

[24] Chiriac H., Corodeanu S., Ţibu M., Óvári T.A. (2007). Size triggered change in the magnetization mechanism of nearly zero magnetostrictive amorphous glass-coated microwires. J. Appl. Phys..

[25] Zhukov A. (2006). Design of the magnetic properties of Fe-rich, glass-coated microwires for technical applications. Adv. Funct. Mater..

[26] Zhukov A., Ipatov M., Churyukanova M., Talaat A., Blanco J.M., Zhukova V. (2017). Trends in optimization of giant magnetoimpedance effect in amorphous and nanocrystalline materials. J. Alloys Compd..

